# Bridging the Gap between Theory and Practice; the Active Role of Inpatient Pharmacists in Therapeutic Drug Monitoring

**DOI:** 10.3390/pharmacy7010020

**Published:** 2019-02-18

**Authors:** Abrar F Alhameed, Sara Al Khansa, Hani Hasan, Sherine Ismail, Mohammed Aseeri

**Affiliations:** 1 King Abdullah International Medical Research Center, King Saud bin Abdulaziz University for Health Sciences, 21423 Jeddah, Saudi Arabia; alhameedab@ngha.med.sa (A.F.A.); Khansasa@ngha.med.sa (S.A.K.); HasanHI@ngha.med.sa (H.H.); EsmailSS@ngha.med.sa (S.I.); 2 Pharmaceutical Care Services, Prince Mohammed Bin Abdulaziz Hospital, MNGHA, 42221 Madinah, Saudi Arabia; 3 Pharmaceutical Care Services, King Khalid Hospital, MNGHA, 21589 Jeddah, Saudi Arabia

**Keywords:** drug monitoring, vancomycin, aminoglycosides, TDM, inpatient, pharmacist

## Abstract

Many hospitals face barriers in the implementation of TDM services, this study aimed to evaluate a pharmacist-led TDM service to optimize patients’ outcomes. Adult patients who were administered vancomycin, gentamicin, or amikacin were included. The pre-phase included a retrospective assessment of patients and the intervention phase consisted of an educational program. The post-phase assessed patients based on TDM services provided by inpatient pharmacists on a 24-h, 7-day basis for 3 months. The primary outcome was to assess the mean difference in proportion of correct initial doses of prescribing orders. Secondary outcomes included assessing the mean differences in proportions of correct dose adjustments and correct drug sampling time. Seventy-five patients in each phase were eligible. Patients who received optimal initial dosing in the post-phase showed a higher statistical significance, mean difference of 0.31, [95% CI (0.181–0.4438), *p* < 0.0001]. Patients in the post-phase received more optimal dose adjustments, mean difference of 0.1, [95% CI (−0.560–0.260), *p* = 0.2113]. Drug levels were ordered more correctly in the post-phase, mean difference of 0.03, [95% CI (−0.129–0.189), *p* = 0.7110]. This study demonstrated the important role of TDM services led by pharmacists in optimizing the initial dosing for these antibiotics.

## 1. Introduction

Therapeutic drug monitoring is a fundamental responsibility of pharmacists to provide optimum therapeutic outcomes for patients [1]. Therapeutic drug monitoring (TDM) is defined as measuring concentrations of certain drugs at specific times to maintain a steady state concentration in the blood and subsequently individualize dosing regimens to achieve target therapeutic goals [1,2]. Pharmacist-led TDM services have demonstrated positive outcomes which include decreased incidence of adverse effects of drug therapy, reduced length of treatment, reduced length of hospital stay, decreased morbidity, decreased mortality, and cost-savings [1,2,3,4,5,6,7]. The most commonly monitored drugs by pharmacists were vancomycin and aminoglycosides [8,9].

Although evidence has demonstrated the benefits of these pharmaceutical services, many healthcare settings have not been able to optimally utilize the knowledge and skills of inpatient pharmacists to provide advanced patient-centered services [4,10]. In a recent study done by Kheir et al. (2015), barriers for providing TDM services have been identified, which include pharmacists spending most of their time on dispensing medications and inventory issues rather than direct patient-care services, lack of practical knowledge to implement the basics of pharmacokinetic (PK) principles to provide effective TDM services, and lack of PK-related continued education topics and training [4]. The study also mentioned that according to observations, pharmacokinetic services are mostly performed by healthcare practitioners rather than pharmacists in most hospitals. 

In Saudi Arabia, published studies assessing hospital pharmacy practices and TDM services are very limited. According to a recent national study done by Alsultan et al. (2013), which described hospital pharmacy practice in Riyadh, Saudi Arabia, only 41% of hospitals had pharmacists routinely monitor serum medication concentrations or their surrogate markers to evaluate drug therapy outcome and toxicity. This is considerably lower than the rate reported by the American Society of Health-System Pharmacists (ASHP) survey in 2010, where more than 92% of hospitals were engaged in this activity [11,12].

The need to provide training programs or continuing professional development to update and standardize the pharmacists’ knowledge and skills in clinical PK has been proposed in a recent study [4]. However, knowledge must be followed by application and translation into clinical practice. The ASHP identified eight general responsibilities of all pharmacists regarding pharmacokinetic monitoring [1]. However, current practice has shown that the inpatient pharmacists in our institution practiced only 2–3 of these responsibilities. 

In our hospital setting, clinical pharmacists routinely follow-up patients during their rounds and provide TDM services, however, this service is limited to day shifts and weekdays only. In addition, TDM services are dependent on consultations by the primary medical team, which may lead to missing patients who require TDM services. This is a practice gap, which there could be improved by involving inpatient pharmacists. However, the transition of newly hired pharmacists from the outpatient pharmacy to the inpatient pharmacy does not involve any formal training for implementing TDM services. The competencies of inpatient pharmacists can be further enhanced by the provision of educational lectures, training, and tools including flowcharts and screening checklists [13].

The aim of this study was to evaluate the impact of an inpatient pharmacist-led service to improve therapeutic drug monitoring of antibiotics and to fill-in the gaps by enhancing pharmacist training regarding TDM, and providing 24-h services on a daily basis focusing on vancomycin and aminoglycosides with the goal of optimizing patient care at our institution. 

## 2. Materials and Methods 

### 2.1. Study Design 

The study design was quasi-experimental, and it was conducted in a tertiary care hospital in Jeddah.

In our hospital, clinical pharmacists attend daily rounds with the medical teams and make recommendations related to optimization of drug therapy, and provide daily follow-up for patients to achieve target therapeutic outcomes. In addition, clinical pharmacists have other drug-related responsibilities such as participating as active members in the P & T Committee, medication-safety, ADR Committee, developing drug protocols, etc. They are also responsible for training interns and residents and conducting clinical research, however, they are not responsible for the verification of prescribed medication orders.

On the other hand, inpatient pharmacists are primarily in-charge of electronic verification of prescribed medication orders, providing medication counseling, and reconciliation. They do not provide routine follow-up of patients to achieve their therapeutic goals. Furthermore, they don’t attend daily rounds with the medical team.

Eligible patients in the study were adults (>18 years of age) who received vancomycin, gentamicin, or amikacin intravenously. Those who received antibiotics for surgical prophylaxis, and patients in the intensive care unit or cardiac intensive care unit were excluded. The study consisted of three phases: pre-phase, intervention phase, and post-phase. The pre-phase included a retrospective assessment of all eligible patients for 3 months by a pharmacy resident. In the intervention phase, the pharmacy resident provided pharmacokinetics interactive sessions for 2 weeks to a team of inpatient pharmacists. These sessions were supervised by four clinical pharmacists and included flowcharts, checklists for dosing and monitoring of vancomycin and aminoglycosides, and provided assessment questions. These were based on the hospital guidelines, which have been approved by the corporate P & T, which was in accordance with the ASHP/IDSA guidelines [14] and drug monographs. Pharmacists’ performance on assessment questions with a maximum score of 100 points was conducted before and after the educational sessions. The post-phase was based on pharmacists providing TDM services for the 3 antibiotics on a 24-h, 7 day basis for 3 months. TDM services by inpatient pharmacists included providing recommendations to the physicians or clinical pharmacists regarding: initial dose, dose adjustment, and laboratory drug level requests. Therapeutic interventions, follow-up, lab results, and endorsements were documented in sheets and kept in one file, which was shared with the next on-duty pharmacist to facilitate the communication. This study received the approval of the Institutional Review Board of King Abdullah International Medical Research Center. 

The primary outcome was to assess the mean difference in proportion of correct initial doses of prescribing orders for these antibiotics as per hospital guidelines before and after intervention. Secondary outcomes included assessing the mean difference in proportions of correct dose adjustment orders and correct orders for drug sampling time within 8 h of the intervention. The orders were considered appropriate if they were corrected within the pharmacist shift (8 h). 

### 2.2. Sample Size 

A sample of 75 patients per treatment group was estimated to provide a 90% power to detect a difference of 25% between two phases for the proportions of the correct initial doses of prescribing orders [15]. A list was provided by the Information Technology (IT) department in which all eligible patients were included by convenience sampling.

### 2.3. Statistical Analysis 

Descriptive statistics were used as deemed necessary for baseline characteristics, initial doses of prescribing orders, dose adjustment orders, and orders for sampling time. The Mann–Whitney test was used to compare non-normally distributed continuous baseline characteristics. Chi-square and Fischer’s exact tests were used if cell count was 5 or less for binary and categorical baseline demographics, initial doses of prescribing orders, dose adjustment orders, and orders for drug sampling time. Two-sample tests of proportions were used to determine the mean difference of proportions and 95% confidence interval for the initial doses of prescribing orders, dose adjustment orders, and orders for sampling time. We used two-sided tests and a *p*-value of 0.05, and 95% confidence intervals were used as cut off level for significance in all analyses. Analyses were performed using Excel® (for Mac 2011 Version 14.7.2) and STATA 14 (StataCorp LP, College Station, TX, USA). 

## 3. Results

**Figure 1:**
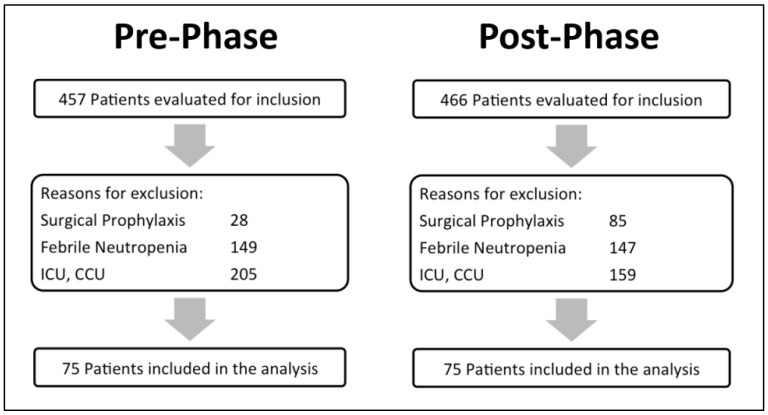
Patient’s enrollment.

A total of 923 patients were screened for eligibility, 457 patients in the pre-phase and 466 in the post-phase, of which 75 patients were included in each phase (Figure 1; patient ‘s enrollment). There were no statistically significant differences in the baseline characteristics between the two groups. Vancomycin was the most commonly prescribed antibiotic (95%) compared to aminoglycosides. Most of the antibiotics used started in the emergency department (Table 1; baseline characteristics).

The percentage of patients who received optimal initial dosing of vancomycin or aminoglycosides was significantly higher post implementation of the program (91% versus 60%, respectively) further outcomes showed slightly positive results (Table 2; primary and secondary outcomes).

**Figure 2:**
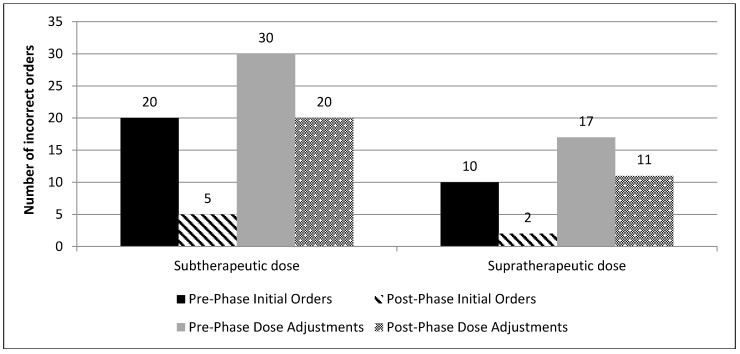
The proportion of subtherapeutic and supratherapeutic initial doses and dose adjustments in both phases.

**Figure 3:**
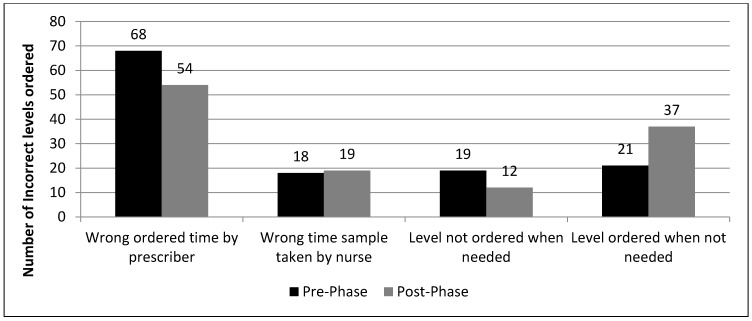
Types of incorrect drug levels ordered.

Incorrect initial orders and dose adjustments were mostly sub-therapeutic in both phases (Figure 2; the proportion of subtherapeutic and supratherapeutic initial doses and dose adjustments in both phases). The most common reason for improper drug levels was the improper order time by the prescriber 68 versus 54, in the pre-phase versus post-phase, respectively (Figure 3; types of incorrect drug levels ordered).

Patients in the post-phase had a less duration of antibiotic therapy with a median of 6 days; Interquartile range (IQR: 4–13) compared to the pre-phase with a median of 8 days; (IQR: 5–13), (*p* = 0.1596).

## 4. Discussion

It has been reported by previous studies that practicing therapeutic drug monitoring according to consensus recommendations of guidelines will more likely lead to target trough levels, which correlate with clinical efficacy [14]. Similar to previous literature, our study shows a higher proportion of patients who received the correct initial dosing, correct dose adjustments and were ordered correct drug levels after the implementation of a pharmacist-led TDM service, our findings are consistent with Marquis et al. (2015) [15]. 

According to two previously published studies, computerized physician order entry (CPOE) had modest effects in optimizing therapeutic drug monitoring of vancomycin [16,17]. First, Damfu et al. (2016) [17] focused only on surgical patients, and the education provided was for surgical residents on how to use a CPOE order set for vancomycin. Although Damfu et al. (2016) [17] was conducted in our setting, the findings were different due to the different target populations between the two studies, and that the interventional phase focused on the inpatient pharmacists. This led to a higher percentage of correct initial drug orders in our study compared to Damfo et al. (2016) [17], which demonstrated that combining CPOE use with pharmacist-led TDM services will most likely achieve target therapeutic goals. 

Marquis et al. (2015) [15] showed a significant improvement in vancomycin initial dosing within 24 h when engaging pharmacists, which resulted in 50% more patients being dosed optimally. However, we measured TDM services as a whole including subsequent dose adjustments and drug levels, which is essential for the continuum of care and to achieve target therapeutic outcomes for patients. In addition, in their study, a prescription order was considered appropriate if corrected within 24 h vs. 8 h in our study, which focuses on an earlier correction of orders. We chose a narrow interval in our study, as time is crucial for dosing newly started patients on antibiotics and to highlight the crucial impact of inpatient pharmacist’s intervention to fill in the gaps when the clinical pharmacist is not on duty. In addition, Marquis et al. (2015) [15] reported 40% correct orders within 24 h compared to 60% within 8 h in our hospital.

Regarding the initial dosing, we found a statistical significant mean difference between the two phases as the pharmacists might focus more on the first time the patient received an antibiotic by carefully assessing all clinical parameters. For dose adjustments, there was an improvement in the post-phase compared to the pre-phase, however, it did not reach statistical significance. A major contributing factor to these findings is the time required to follow up patients on subsequent days in case of any changes in the clinical condition of the patients (e.g., change in renal function), which is beyond the scope of the pharmacists’ usual daily practice (order verification in our setting). Daily TDM services and follow-up require protected time from pharmacists to re-evaluate the clinical status of patients and make subsequent changes. 

Regarding drug levels, an important finding in our study is that there were more total drug level orders requested in the post-phase than in the pre-phase. The number of drug levels ordered unnecessarily was also more in the post-phase, 37 vs. 21 (Figure 3), which may lead to wastage of resources. This is most likely due to the fact that medical residents/physicians know more about the use of drug levels but not the proper sampling time and frequency, hence the pharmacist or clinical pharmacist may need to request additional orders for proper drug levels. 

There are several limitations to our study. First, limited generalizability because vancomycin prescription orders were the highest among all ordered antibiotics, so expanding the findings of our results to aminoglycosides may not be appropriate. Second, inpatient pharmacists cannot change a medication order without the approval of the prescriber, which might have underestimated the correct orders suggested by pharmacists to implement a dose change if the prescriber did not approve it or respond to pharmacist’s call. Third, it was difficult for pharmacists to correct or cancel inappropriate ordered drug levels as they were usually ordered without a notification by the system to the pharmacists, since these are not medication orders. Fourth, the study is a quasi-experimental with two different populations before and after which might have introduced different types of bias such as maturation and instrumentation, due to the different skills among inpatient pharmacists in both study phases, which may affect internal validity. Fifth, the limited number of participating pharmacists which might have diluted the impact of TDM services and subsequent dosage adjustments due to the difficulty of following-up patients if other untrained pharmacists were in service. 

Our study has several strengths. First, the quasi-experimental design is a useful tool to assess practice changes after implementation of a new intervention; second, we were able to assess the three main components of TDM services; and third, we were able to engage inpatient pharmacists in patient-centered clinical services. To our knowledge, this is the first study in Saudi Arabia to assess inpatient pharmacists’ involvement in therapeutic drug monitoring of vancomycin and aminoglycosides.

Future studies shall explore implementing CPOE and educational phases together, to assess the outcomes of practicing collaborative-practice models which empower inpatient pharmacists with protected time and full TDM privileges, including dose adjustments and drug level ordering. 

## 5. Conclusions

This study shows the importance of a TDM-led service by pharmacists, which had a positive impact on optimizing the initial dosing for vancomycin, amikacin, and gentamicin. However, there is a need for future studies to explore opportunities for pharmacy practice models addressing barriers to optimize dose adjustments and lab monitoring for TDM services. 

## Figures and Tables

**Table 1:** Baseline characteristics.

	Pre-Phase (n = 75)	Post-Phase (n = 75)	*p*-Value ^a^
	n (%) or Median; IQR		
Age (years)	66 (49–79)	63 (51–77)	0.7607
Sex (male)	38 (51%)	38 (51%)	1
Body Mass Index (kg/m^2^)	24.8 (20.6–30.8)	25.3 (21–31.2)	0.3904
Patients on dialysis	7 (9.33%)	13 (17.33%)	0.150
Prescribed antibiotic			
Vancomycin	71/75 (95%)	71/75 (95%)	1
Gentamicin	4/75 (5%)	3/75 (4%)	1
Amikacin	0/75 (0%)	1/75 (1.3%)	1
Baseline lab values at the time of initiation of antibiotic			
CrCl (mL/min) ^b^	67.9 (37.3–106.5)	60 (30–94)	0.3082
WBCs (×10^9^ cells/L)	10.9 (7.6–16.1)	11 (7.7–16)	0.7042
Wards at which antibiotics were initiated			
Emergency	36 (48%)	35 (46.6%)	0.87
Medical	23 (30.7%)	32 (42.6%)	0.127
Surgical	16 (21.3%)	8 (10.6%)	0.075
Indications			
Skin and Soft Tissue	5 (6.6%)	5 (6.6%)	1
Bacteremia	24 (32%)	35 (46.6%)	0.066
Osteomyelitis	6 (8%)	3 (4%)	0.494
Pneumonia	20 (26.6%)	16 (21.3%)	0.472
Endocarditis	0 (0%)	1 (1.3%)	1
Meningitis	8 (10.6%)	4 (5.3%)	0.367
Urinary Tract Infection	8 (10.6%)	7 (9.3%)	0.785
Intra-abdominal infection	1 (1.3%)	4 (5.3%)	0.367
Other ^c^	3 (4%)	0 (0%)	0.367

Abbreviations: SrCr: serum creatinine; CrCl: creatinine clearance; WBC: white blood cells; IQR: Interquartile range, kg/m^2^: kilogram/meter^2^, cells/L: cells per liter; a: Mann–Whitney test for continuous variables, and Chi-square or Fisher’s exact tests for proportions as deemed necessary; b: based on the Cockcroft and Gault equation; c: endophthalmitis, rhinosinusitis, and nasal Methicillin-resistant staphylococcus aureus.

**Table 2:** Primary and secondary outcomes.

Outcome	Pre-Phase	Post-Phase	Mean Difference (95% Confidence Interval)	*p*-Value
Optimal initial dosing	60 % (45/75)	91% (68/75)	0.31 (0.18–0.44)	<0.0001
Optimal dose adjustments	55 % (61/111)	65% (52/80)	0.1 (−0.05–0.26)	0.2113
Optimal drug level requests	55 % (153/279)	58% (171/293)	0.03 (−0.13–0.19)	0.7110
